# Supplementary material for: Tumor-Specificity, Neurotoxicity, and Possible Involvement of the Nuclear Receptor Response Pathway of 4,6,8-Trimethyl Azulene Amide Derivatives
Source: Int J Mol Sci. 2022 Feb 26;23(5):2601. doi: 10.3390/ijms23052601 (PMC8910578; doi:10.3390/ijms23052601)
Supplement: Supplementary file 1 [file ijms-23-02601-s001.zip › ijms-1606747-supplementary.pdf]

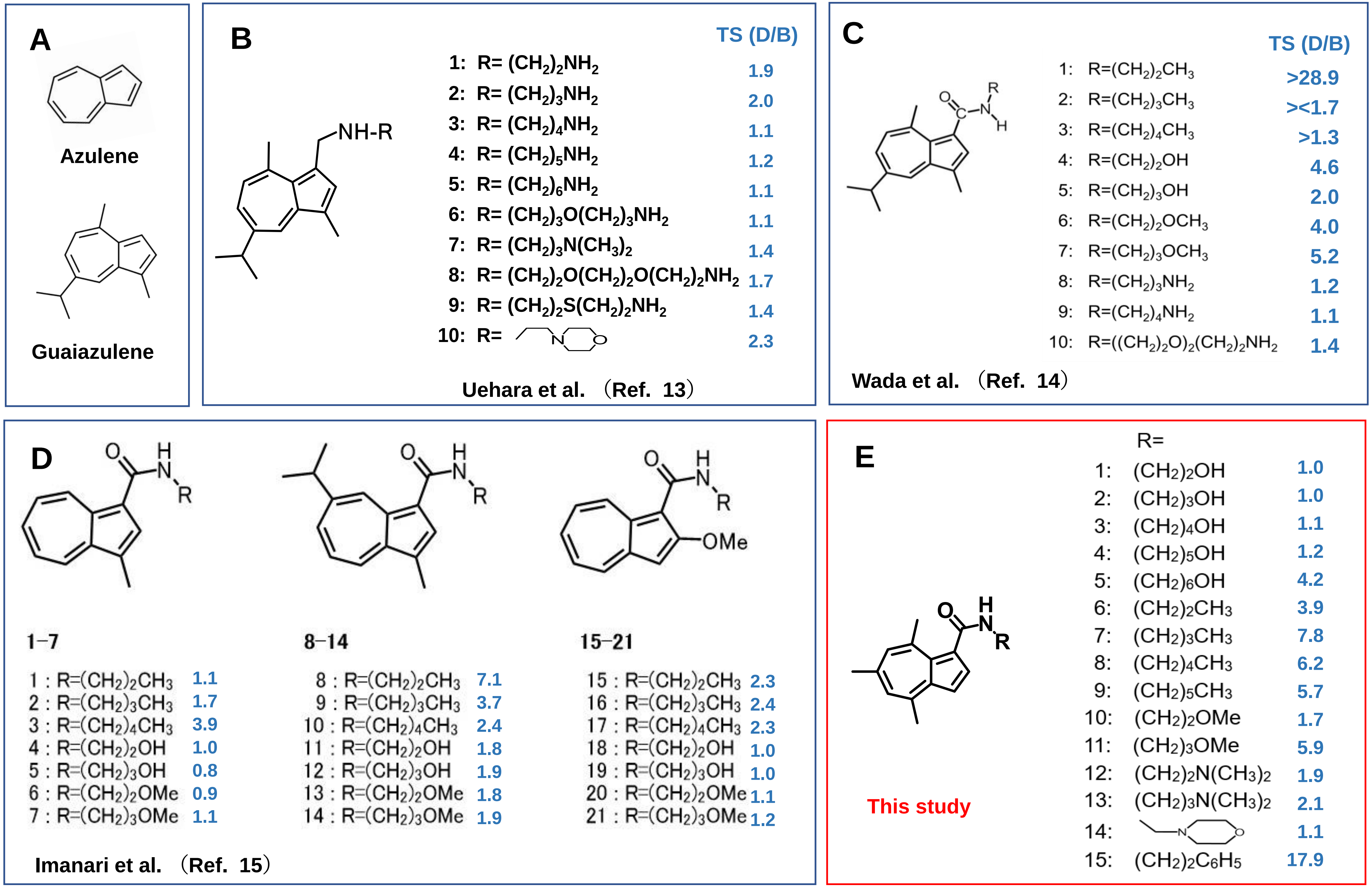

Figure S1

Structures of guaiazulene derivatives in our previous reports (B, C, D) and this study (E).

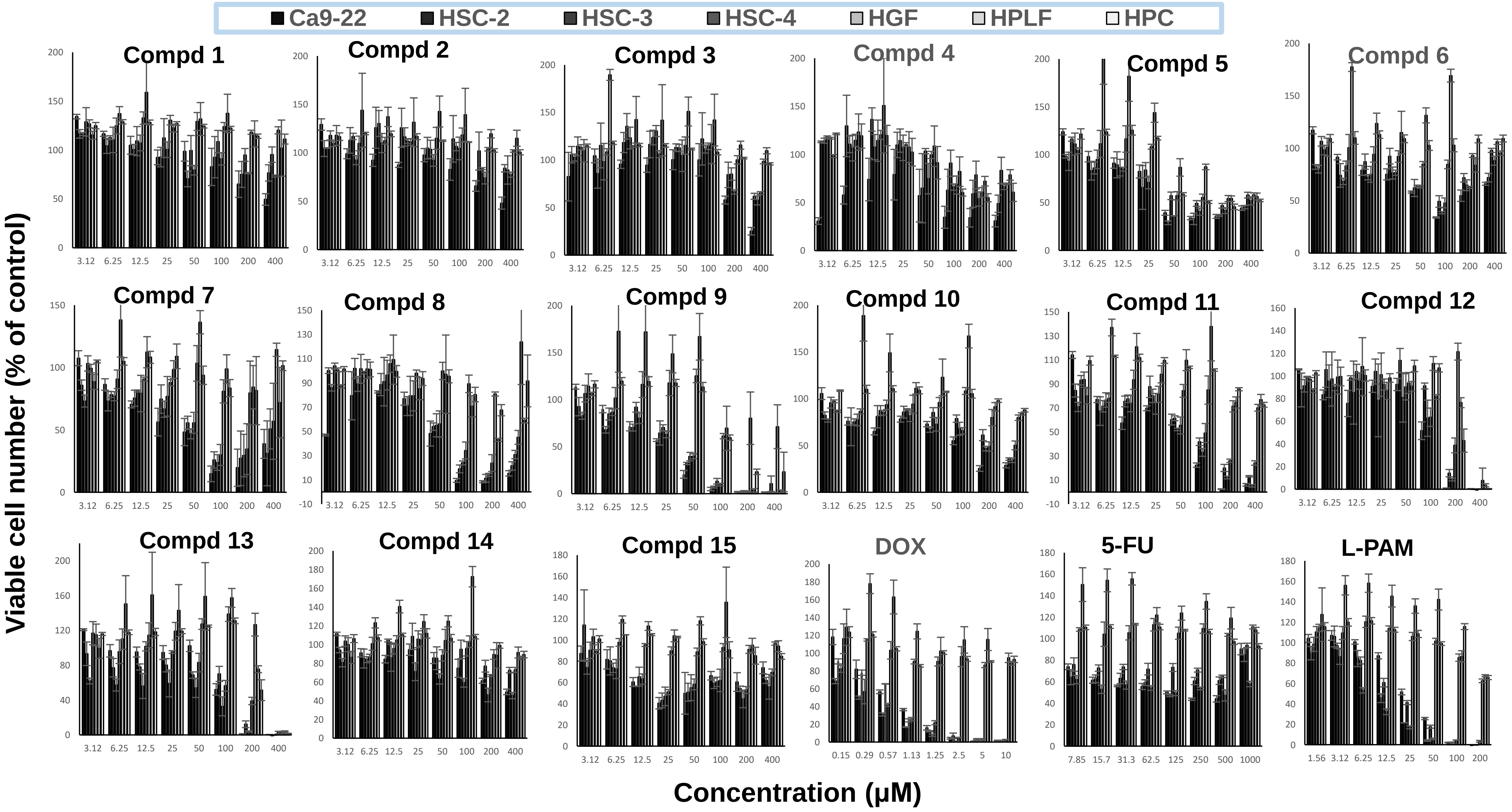

Figure S2 (1st experiment of dose-response of compounds 1-10, DOX, 5-FU and L-PAM

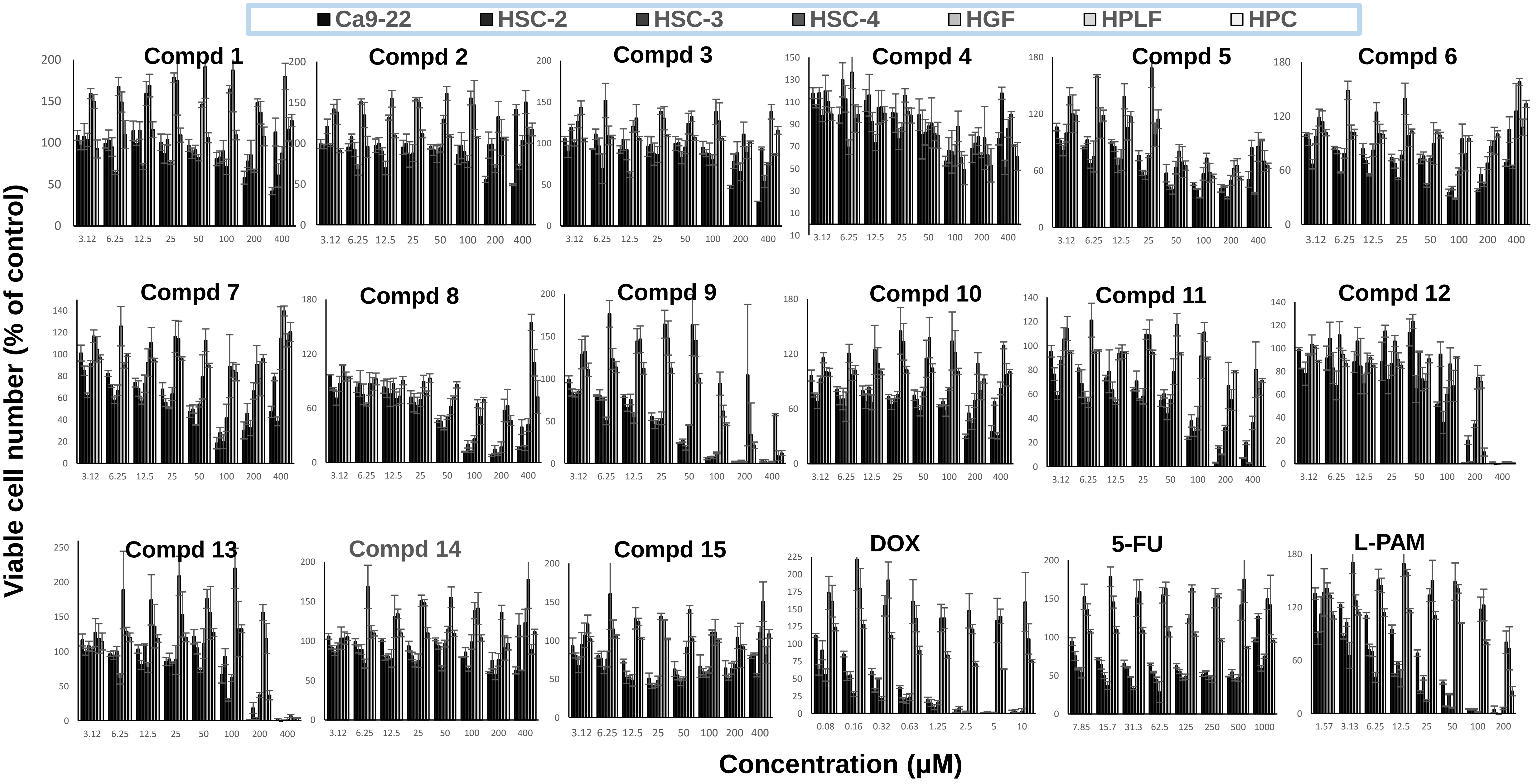

Figure S3 (2nd experiment of dose-response of compounds 1-10, DOX, 5-FU and L-PAM

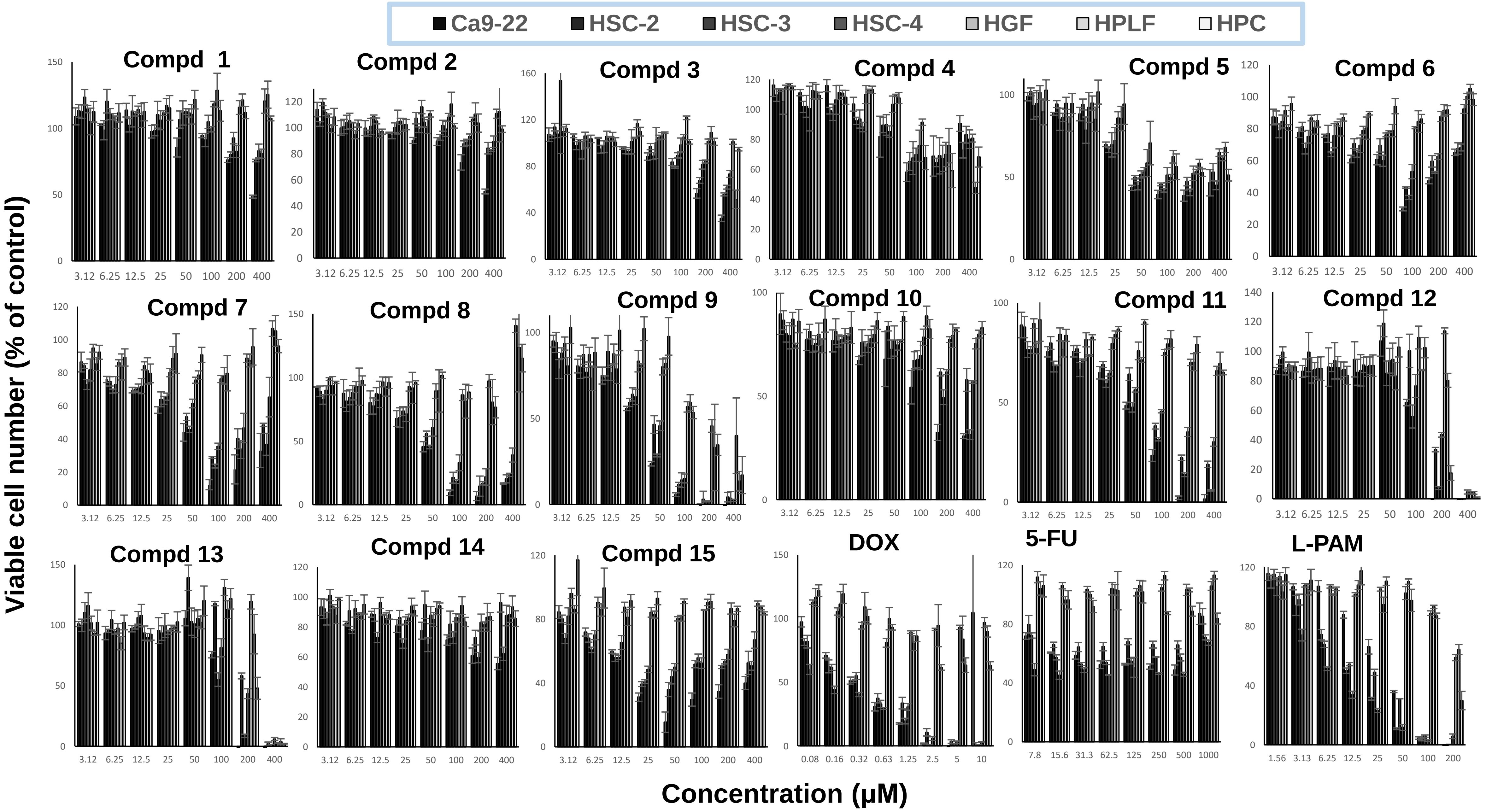

Figure S4 (3rd experiment of dose-response of compounds 1-10, DOX, 5-FU and L-PAM

Sampling: 20210121   Applied protein: 15 μg

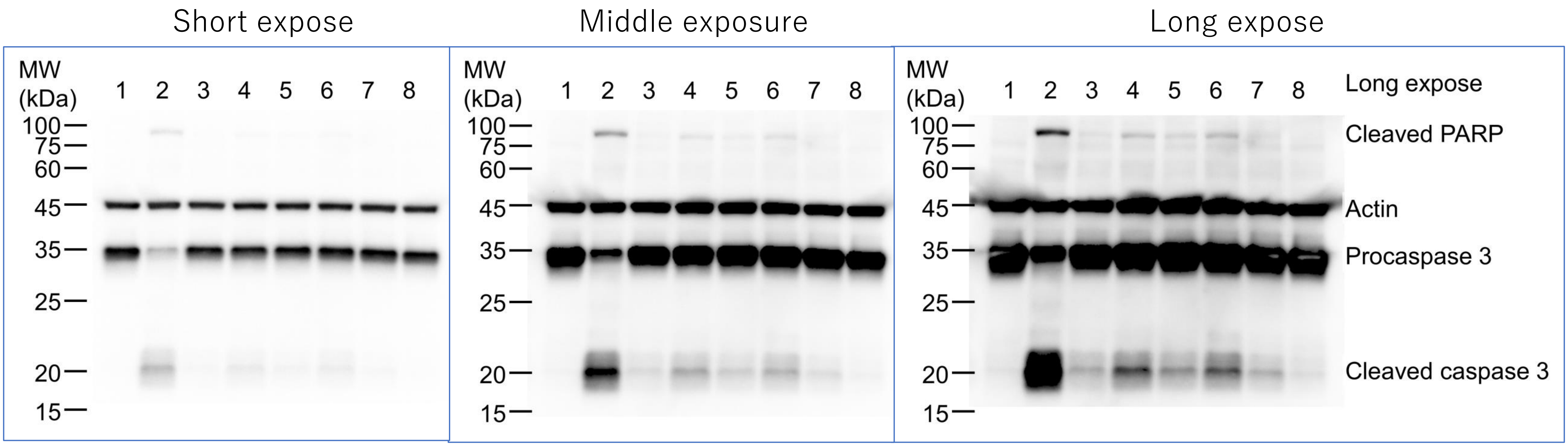

Figure S5. Raw data of stained gel and images after short and long exposure (without and with contrast adjustment)

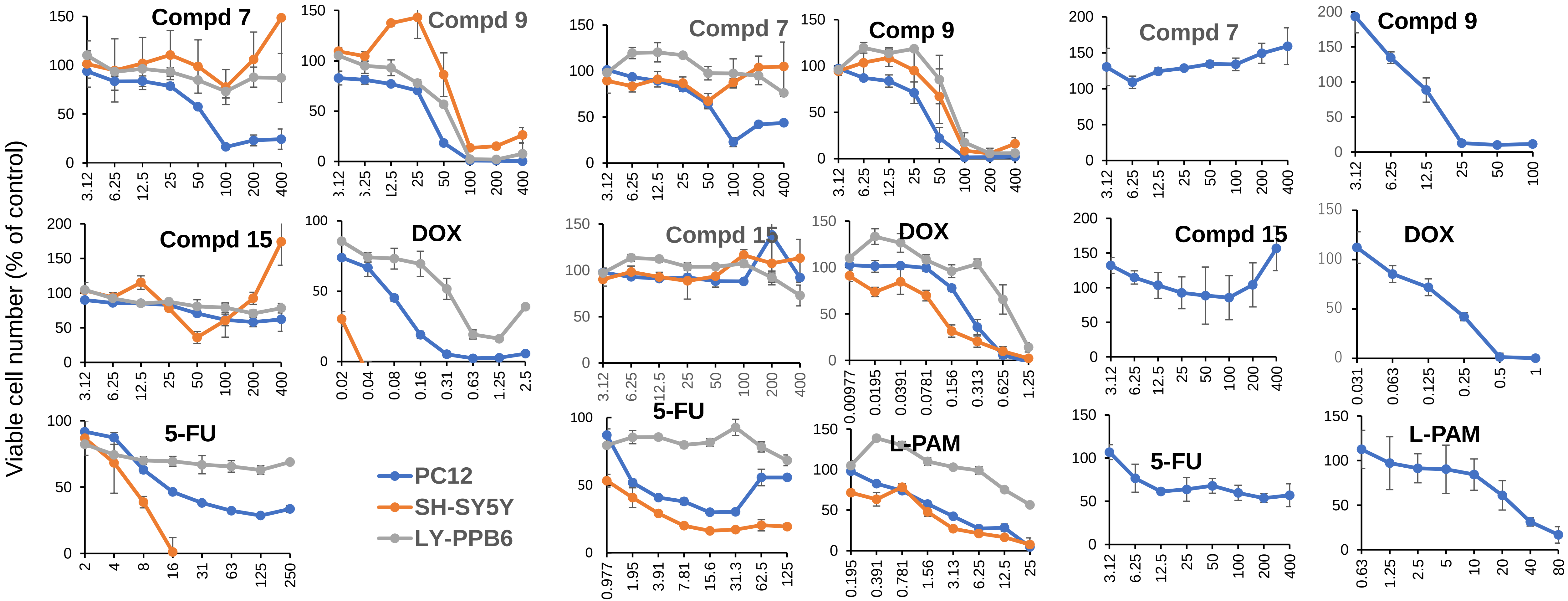

A	CC50 (μM)					
	PC12	SH-SY5Y	LYPP B6	mean (E)	Normal cells (D)	NT (D/E)
**7**	59	400	400	286	386	1.4
**9**	35	73	56	55	189	3.5
**15**	378	42	400	273	393	1.4
DOX	0.07	0.02	0.32	0.14	9.68	70.6
5-FU	14	5.85	250	89.95	1000	11.0

B	CC50 (μM)					
	PC12	SH-SY5Y	LYPP B6	mean (E)	Normal cells (D)	NT (D/E)
**7**	68	400	400	289	386	1.3
**9**	36	63	74	58	189	3.3
**15**	400	400	400	400	393	1.0
DOX	0.26	0.12	0.78	0.39	10	25.0
5-FU	2	1	125	43	1000	23.3
L-PAM	2	2	25	10	191	19.8

C	CC50 (μM)		
	Diff PC12 (F)	Normal cells (D)	NT (D/F)
7	400	386	1.0
9	19	189	10.2
15	400	393	1.0
DOX	0.22	10	44.9
5-FU	297	1000	3.4
L-PAM	26	191	7.2

Figure S6 Neurotoxicity of compounds 7, 8 and 9 in comparison with reference compounds

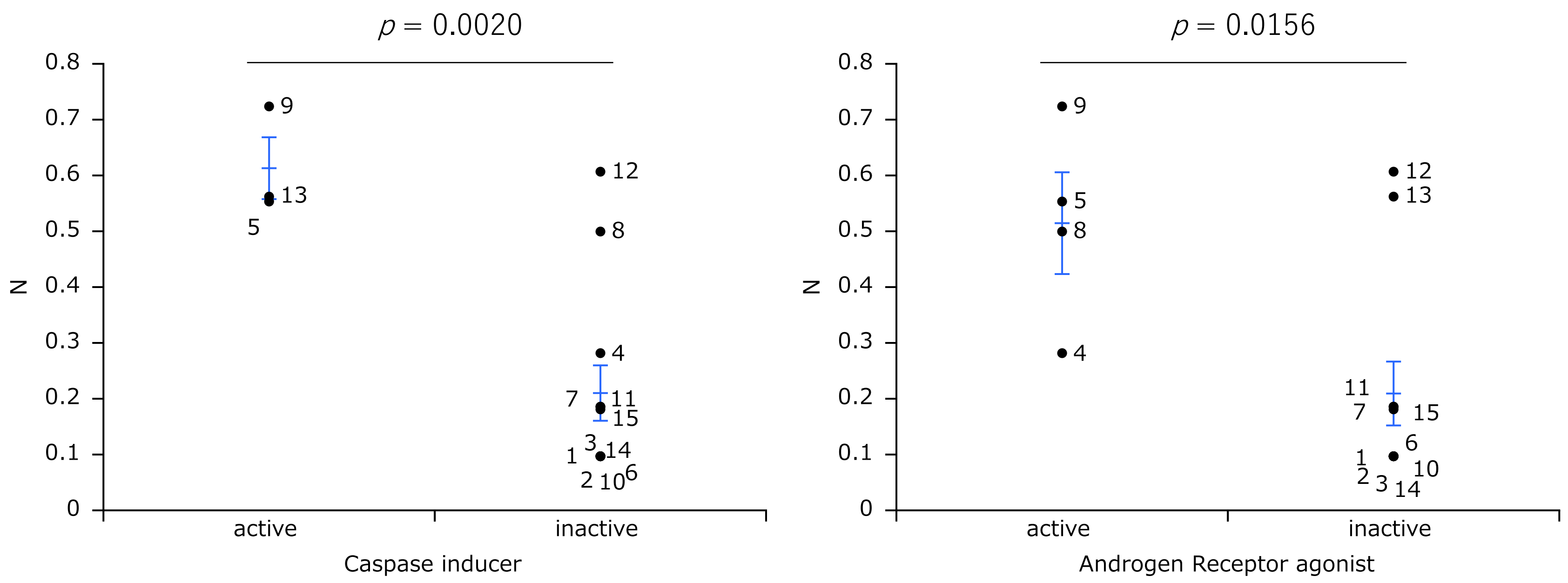

Figure S7 Nuclear receptors and stress response pathways selectively associated with cytotoxicity against normal oral cells. The t-test was performed for cytotoxicity against humal normal oral cells (N) between azlene derivatives predicted as active and inactive in each biochemical pathway.

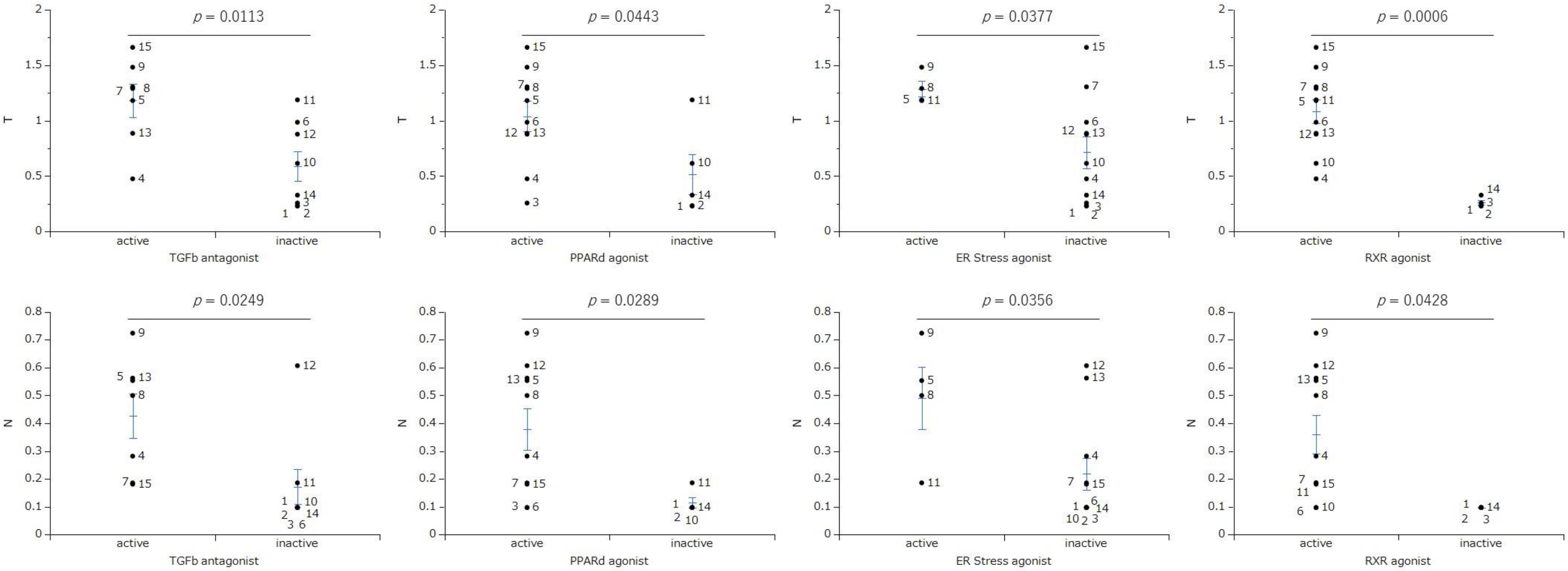

Figure S8 Nuclear receptors and stress response pathways non-selectively associated with cytotoxicity against both normal oral cells and oral squamous cell carcinoma cell lines. The *t*-test was performed for cytotoxicity against both normal and tumor cytotoxicity (N, T) between azulene derivatives predicted as active and inactive in each biochemical pathway.TGFb, PPARd, ER Stress, and RXR are transforming growth factor β, peroxisome proliferator activated receptor δ, endoplasmic reticulum stress, retinoid X receptor, respectively.

Exp. 1

	CC50 (μM): Human oral squamous cell carcinoma cell lines						CC50 (μM): Human normal oral cells					TS		PSE	
	Ca9-22 (A)	HSC-2	HSC-3	HSC-4	mean (B)	SD	HGF (C)	HPLF	HPC	mean (D)	SD	(D/B)	(C/A)	$(100D/B^2)$	$(100C/A^2)$
1	327.7	400.0	400.0	400.0	381.9	36.2	400.0	400.0	400.0	400.0	0.0	1.0	1.2	0.3	0.4
2	363.7	333.3	400.0	400.0	374.3	32.2	400.0	400.0	400.0	400.0	0.0	1.1	1.1	0.3	0.3
3	247.3	400.0	400.0	333.3	345.2	72.4	400.0	400.0	400.0	400.0	0.0	1.2	1.6	0.3	0.7
4	64.4	141.4	400.0	326.7	233.1	156.5	400.0	400.0	400.0	400.0	0.0	1.7	6.2	0.7	9.6
5	43.9	32.4	111.5	39.9	56.9	36.7	400.0	400.0	115.1	305.0	164.5	5.4	9.1	9.4	20.8
6	66.4	87.9	77.3	90.7	80.6	11.1	400.0	400.0	400.0	400.0	0.0	5.0	6.0	6.2	9.1
7	36.3	59.6	46.0	59.0	50.2	11.2	400.0	270.7	400.0	356.9	74.7	7.1	11.0	14.2	30.4
8	49.2	55.7	56.7	62.2	55.9	5.3	400.0	172.3	400.0	324.1	131.4	5.8	8.1	10.4	16.5
9	29.7	34.4	41.7	39.1	36.2	5.3	400.0	126.1	126.3	217.5	158.1	6.0	13.5	16.6	45.3
10	117.3	283.7	183.7	186.0	192.7	68.5	400.0	400.0	400.0	400.0	0.0	2.1	3.4	1.1	2.9
11	63.0	79.6	55.3	92.0	72.5	16.5	400.0	400.0	400.0	400.0	0.0	5.5	6.3	7.6	10.1
12	105.3	154.0	118.7	151.0	132.3	24.0	326.0	272.3	190.7	263.0	68.1	2.0	3.1	1.5	2.9
13	105.2	134.0	34.6	133.3	101.8	46.8	322.3	270.0	215.3	269.2	53.5	2.6	3.1	2.6	2.9
14	373.3	333.3	195.0	400.0	325.4	91.2	400.0	400.0	400.0	400.0	0.0	1.2	1.1	0.4	0.3
15	19.1	19.8	55.3	24.3	29.6	17.3	400.0	400.0	333.3	377.8	38.5	12.8	20.9	43.0	109.6
DOX	0.41	0.16	0.43	0.20	0.30	0.14	10.00	7.08	10.00	9.03	1.68	30.0	24.4	9986.4	5929.5
5-FU	124.0	109.0	1000.0	72.6	326.4	449.6	1000.0	1000.0	1000.0	1000.0	0.0	3.1	8.1	0.9	6.5
L-PAM	27.1	11.9	19.3	9.3	16.9	8.0	200.0	200.0	200.0	200.0	0.0	11.8	7.4	70.2	27.3

Exp. 2

	CC50 (μM): Human oral squamous cell carcinoma cell lines						CC50 (μM): Human normal oral cells					TS		PSE	
	Ca9-22 (A)	HSC-2	HSC-3	HSC-4	mean (B)	SD	HGF (C)	HPLF	HPC	mean (D)	SD	(D/B)	(C/A)	$(100D/B^2)$	$(100C/A^2)$
1	287.3	400.0	392.3	400.0	369.9	55.2	400.0	400.0	400.0	400.0	0.0	1.1	1.4	0.3	0.5
2	376.7	400.0	400.0	400.0	394.2	11.7	400.0	400.0	400.0	400.0	0.0	1.0	1.1	0.3	0.3
3	194.0	400.0	400.0	400.0	348.5	103.0	400.0	400.0	400.0	400.0	0.0	1.1	2.1	0.3	1.1
4	400.0	400.0	283.0	400.0	370.8	58.5	400.0	400.0	400.0	400.0	0.0	1.1	1.0	0.3	0.3
5	72.8	37.7	33.7	221.7	91.5	88.6	400.0	291.7	200.0	297.2	100.1	3.2	5.5	3.6	7.5
6	80.3	86.3	28.4	400.0	148.7	169.5	400.0	400.0	400.0	400.0	0.0	2.7	5.0	1.8	6.2
7	47.2	49.5	26.6	57.7	45.2	13.2	400.0	400.0	400.0	400.0	0.0	8.8	8.5	19.5	18.0
8	46.3	43.1	36.4	49.1	43.7	5.5	264.7	228.2	184.0	225.6	40.4	5.2	5.7	11.8	12.3
9	28.7	22.8	25.0	22.7	24.8	2.8	331.3	174.7	96.5	200.8	119.6	8.1	11.5	32.6	40.1
10	140.7	327.3	157.3	400.0	256.3	127.6	400.0	400.0	400.0	400.0	0.0	1.6	2.8	0.6	2.0
11	57.1	72.4	38.3	73.7	60.4	16.6	400.0	266.7	400.0	355.6	77.0	5.9	7.0	9.8	12.3
12	103.7	159.7	74.5	137.0	118.7	37.4	267.3	261.0	151.7	226.7	65.0	1.9	2.6	1.6	2.5
13	123.0	156.7	76.5	145.7	125.5	35.5	342.0	316.0	186.3	281.4	83.4	2.2	2.8	1.8	2.3
14	400.0	400.0	333.3	400.0	383.3	33.3	400.0	400.0	400.0	400.0	0.0	1.0	1.0	0.3	0.3
15	24.0	14.0	15.5	16.6	17.5	4.4	400.0	400.0	400.0	400.0	0.0	22.8	16.7	130.2	69.6
DOX	0.5	0.2	0.3	0.1	0.2	0.2	10.0	10.0	10.0	10.0	0.0	40.7	22.1	16524.6	4901.9
5-FU	314.0	1000.0	20.4	9.5	336.0	464.6	1000.0	1000.0	1000.0	1000.0	0.0	3.0	3.2	0.9	1.0
L-PAM	38.7	11.0	17.5	4.9	18.0	14.7	200.0	200.0	155.0	185.0	26.0	10.3	5.2	56.9	13.3

Exp.3

	CC50 (μM): Human oral squamous cell carcinoma cell lines						CC50 (μM): Human normal oral cells					TS		PSE	
	Ca9-22 (A)	HSC-2	HSC-3	HSC-4	mean (B)	SD	HGF (C)	HPLF	HPC	mean (D)	SD	(D/B)	(C/A)	$(100D/B^2)$	$(100C/A^2)$
1	390	400	400	400	398	4.8	400	400	400	400	0.0	1.0	1.0	0.3	0.3
2	398	400	400	400	399	1.2	400	400	400	400	0.0	1.0	1.0	0.3	0.3
3	259	400	400	400	365	70.5	400	400	400	400	0.0	1.1	1.5	0.3	0.6
4	400	400	400	400	400	0.0	400	375	331	368	35.1	0.9	1.0	0.2	0.3
5	44	52	44	58	50	6.8	80	400	231	237	160.1	4.8	1.8	9.6	4.2
6	66	87	71	100	81	15.3	400	400	400	400	0.0	4.9	6.0	6.1	9.1
7	41	56	44	72	53	14.0	400	400	400	400	0.0	7.5	9.7	14.0	23.3
8	45	58	46	69	55	11.6	400	400	400	400	0.0	7.3	8.9	13.5	20.0
9	29	44	35	44	38	7.3	163	168	115	149	29.4	3.9	5.5	10.2	18.9
10	115	400	203	400	279	143.9	400	400	400	400	0.0	1.4	3.5	0.5	3.0
11	48	77	45	79	62	18.3	400	400	400	400	0.0	6.4	8.4	10.3	17.7
12	123	174	111	179	147	34.8	316	279	162	252	80.7	1.7	2.6	1.2	2.1
13	133	229	110	182	163	52.9	319	293	203	272	60.7	1.7	2.4	1.0	1.8
14	400	400	400	400	400	0.0	400	400	400	400	0.0	1.0	1.0	0.3	0.3
15	17	17	17	24	19	3.4	400	400	400	400	0.0	21.5	24.0	115.4	144.6
DOX	0.33	0.34	0.38	0.13	0.29	0.11	10	10	10	10	0.0	34.0	29.9	11536.5	8964.1
5-FU	136	1000	1000	17	538	535.5	1000	1000	1000	1000	0.0	1.9	7.4	0.3	5.4
L-PAM	38	13	23	7	20	13.8	200	200	165	188	20.0	9.3	5.2	45.9	13.7

Mean of Exps 1, 2 and 3

	CC50 (μM): Human oral squamous cell carcinoma					CC50 (μM): Human normal oral cells				TS		PSE	
	Ca9-22 (A)	HSC-2	HSC-3	HSC-4	mean (B)	HGF (C)	HPLF	HPC	mean (D)	(D/B)	(C/A)	$100D/B^2$	$100C/A^2$
1	335.1	400.0	397.4	400.0	383.1	400.0	400.0	400.0	400.0	1.0	1.2	0.3	0.4
2	379.3	377.8	400.0	400.0	389.3	400.0	400.0	400.0	400.0	1.0	1.1	0.3	0.3
3	233.4	400.0	400.0	377.8	352.8	400.0	400.0	400.0	400.0	1.1	1.7	0.3	0.7
4	288.1	313.8	361.0	375.6	334.6	400.0	391.6	376.9	389.5	1.2	1.4	0.3	0.5
5	53.5	40.8	63.2	106.5	66.0	293.3	363.9	181.9	279.7	4.2	5.5	6.4	10.2
6	71.1	86.9	59.0	196.9	103.5	400.0	400.0	400.0	400.0	3.9	5.6	3.7	7.9
7	41.6	55.1	38.9	62.9	49.6	400.0	356.9	400.0	385.6	7.8	9.6	15.7	23.1
8	46.7	52.4	46.3	60.1	51.4	354.9	266.8	328.0	316.6	6.2	7.6	12.0	16.2
9	29.3	33.9	33.8	35.2	33.0	298.2	156.1	112.5	189.0	5.7	10.2	17.3	34.8
10	124.2	337.0	181.3	328.7	242.8	400.0	400.0	400.0	400.0	1.7	3.2	0.7	2.6
11	55.9	76.5	46.3	81.6	65.1	400.0	355.6	400.0	385.2	5.9	7.2	9.1	12.8
12	110.7	162.7	101.3	155.6	132.6	303.2	270.7	168.0	247.3	1.9	2.7	1.4	2.5
13	120.5	173.1	73.6	153.6	130.2	327.7	293.0	201.6	274.1	2.1	2.7	1.6	2.3
14	391.1	377.8	309.4	400.0	369.6	400.0	400.0	400.0	400.0	1.1	1.0	0.3	0.3
15	19.9	16.8	29.4	21.5	21.9	400.0	400.0	377.8	392.6	17.9	20.1	81.7	101.0
DOX	0.4	0.2	0.4	0.1	0.3	10.0	9.0	10.0	9.7	34.5	25.1	12325.0	6270.0
5-FU	191.2	703.0	673.5	33.1	400.2	1000.0	1000.0	1000.0	1000.0	2.5	5.2	0.6	2.7
L-PAM	34.7	11.9	20.1	6.9	18.4	200.0	200.0	173.4	191.1	10.4	5.8	56.5	16.6

Table S1 Cytotoxicity of 4,6,8-7trimethyl azulene amide derivatives and reference compounds against human oral squamous cell carcinoma cell lines and human normal oral cells, and TS and PSE values delineated from CC50 values. The data of 3 independent experiments and mean values are shown.

	r_pearson	r2	p_value
AATSC0v	0.8410942	0.70743946	8.5281E-05
GATS1se	-0.83325773	0.69431845	0.00011431
FASA_H	0.81940379	0.67142257	0.00018535
FASA_P	-0.81940369	0.6714224	0.00018535
GATS4s	0.81774718	0.66871046	0.00019585
vsurf_CW5	-0.81702439	0.66752886	0.00020058
AMID_C	0.81139109	0.6583555	0.00024077
ASA_P	-0.8057693	0.64926417	0.00028722
vsurf_W5	-0.80175046	0.6428038	0.00032473
a_donacc	-0.7961373	0.6338346	0.00038373
vsurf_HL1	-0.79582979	0.63334505	0.0003872
AATS1i	-0.7952043	0.63234989	0.00039434
vsurf_CP	0.79291096	0.62870779	0.00042142
AATSC0p	0.78921951	0.62286744	0.0004682
FNSA5	0.78537572	0.61681502	0.00052132
PEOE_VSA-1	0.78475391	0.6158387	0.00053036
vsurf_W4	-0.77246221	0.59669787	0.00073689
vsurf_CW4	-0.76999267	0.59288871	0.00078536
PEOE_VSA1	-0.76827196	0.59024181	0.00082064
AMID_h	-0.75769968	0.5741088	0.00106666
vsurf_HB5	-0.75683009	0.57279178	0.00108929
GATS1are	-0.75540975	0.57064389	0.00112709
MIC0	-0.74963639	0.56195471	0.00129181
FilterItLogS	-0.74938344	0.56157555	0.00129945
PEOE_VSA6	0.74444567	0.55419936	0.00145601
vsurf_HB4	-0.73927012	0.5465203	0.00163608
SLogP	0.7390612	0.54621146	0.00164371
SlogP	0.73905793	0.54620662	0.00164383
ETA_dAlpha_B	-0.73451808	0.53951681	0.0018168
logP(o/w)	0.72697365	0.52849069	0.0021364
h_logS	-0.72563495	0.52654609	0.00219754
ETA_dPsi_A	-0.72530593	0.52606869	0.00221278
vsurf_A	-0.72465225	0.52512088	0.00224332
Q_VSA_FHYD	0.72191997	0.52116845	0.00237461
Q_VSA_FPOL	-0.72191996	0.52116843	0.00237461
vsurf_CW3	-0.71857731	0.51635335	0.00254354
lip_acc	-0.71843266	0.51614549	0.00255106
ETA_epsilon_2	-0.7176485	0.51501937	0.00259215
logS	-0.71503937	0.51128129	0.00273268
SM1_Dzv	0.71335858	0.50888047	0.00282639
h_logP	0.71042416	0.50470249	0.00299613
a_ICM	-0.7086862	0.50223613	0.00310044
IC0	-0.70868603	0.50223589	0.00310045
SM1_Dzi	-0.70834328	0.50175021	0.00312136

	r_pearson	r2	p_value
vsurf_ID2	-0.70755708	0.50063702	0.00316975
ATSC0c	-0.70674683	0.49949109	0.00322024
ETA_dEpsilo	0.70670776	0.49943586	0.00322269
SM1_Dzare	-0.70016623	0.49023275	0.00365452
AATSC0c	-0.70004773	0.49006682	0.00366275
MAXaasC	0.69791244	0.48708177	0.00381353
vsurf_ID3	-0.6973885	0.48635072	0.00385127
SM1_Dzpe	-0.6968113	0.48554598	0.00389319
vsurf_ID5	-0.69039439	0.47664441	0.00438415
SM1_Dzm	-0.68948565	0.47539046	0.00445747
SM1_DzZ	-0.68944675	0.47533682	0.00446063
SM1_Dzse	-0.68835594	0.4738339	0.00454998
RASA	0.68811721	0.4735053	0.00456972
opr_violation	0.68692287	0.47186303	0.00466951
vsurf_W3	-0.68533991	0.46969079	0.00480443
vsurf_D5	0.68266608	0.46603297	0.00503932
AATSC8i	0.68114863	0.46396345	0.00517661
TopoPSA	-0.68009975	0.46253567	0.00527322
TPSA	-0.68009973	0.46253564	0.00527323
MATS8i	0.67832562	0.46012565	0.00543989
vsurf_ID4	-0.67827204	0.46005296	0.00544498
h_ema	-0.6777943	0.45940512	0.0054906
AATS1se	-0.67716037	0.45854617	0.0055516
vsurf_ID1	-0.67656951	0.45774631	0.00560893
ETA_epsilon_	-0.6749637	0.455576	0.00576711
SM1_Dzp	0.67308754	0.45304684	0.00595637
BIC0	-0.67171144	0.45119626	0.00609827
MID_h	-0.67144934	0.45084422	0.0061256
GATS3c	0.6707835	0.44995051	0.00619545
vsurf_IW5	-0.67064467	0.44976428	0.0062101
ASA_H	0.67011081	0.44904849	0.00626667
Q_VSA_POL	-0.66946078	0.44817773	0.00633609
h_logD	0.66593371	0.4434677	0.00672343
Q_VSA_HYD	0.6655985	0.44302136	0.00676119
vsurf_HB2	-0.66355564	0.44030609	0.00699494
GATS4se	0.66173079	0.43788764	0.00720907
GATS4pe	0.65843307	0.43353411	0.00760909
ATSC8i	0.65780004	0.43270089	0.00768783
AATSC0are	-0.65640788	0.43087131	0.00786325
MIC1	-0.65622248	0.43062795	0.00788685
GATS4are	0.65614381	0.4305247	0.00789688
AATSC0pe	-0.65558933	0.42979736	0.00796785
vsurf_D1	0.65423755	0.42802677	0.00814297
vsurf_IW3	-0.65264669	0.4259477	0.00835292

	r_pearson	r2	p_value
AATSC0Z	-0.65236064	0.4255744	0.00839112
vsurf_D3	0.65139796	0.4243193	0.00852067
vsurf_D4	0.65124973	0.42412622	0.00854075
vsurf_HB3	-0.65112747	0.42396699	0.00855735
AATSC0m	-0.64974086	0.42216318	0.00874734
Q_VSA_FPPC	-0.64915037	0.4213962	0.00882924
vsurf_IW2	-0.64675264	0.41828898	0.009168
chi1_C	0.64667355	0.41818667	0.00917934
AATS1are	-0.64655277	0.41803049	0.00919669
GATS8i	-0.64627844	0.41767582	0.00923618
GATS5s	0.64373621	0.41439631	0.00960851
GATS5c	0.64323961	0.41375719	0.00968259
vsurf_D2	0.64120547	0.41114446	0.0099907
vsurf_ID7	-0.6403085	0.40999497	0.01012897
GATS6c	0.63925736	0.40864997	0.0102929
GATS5are	0.63851263	0.40769838	0.01041029
vsurf_CW1	-0.63410102	0.4020841	0.01112725
SMR_VSA5	0.63404362	0.40201131	0.01113683
vsurf_D6	0.63380662	0.40171084	0.01117643
ATSC0are	-0.63294949	0.40062505	0.01132057
GATS5se	0.63113841	0.39833569	0.0116299
E_ang	-0.63038213	0.39738163	0.01176099
GATS5pe	0.62965638	0.39646716	0.01188788
SIC0	-0.62935612	0.39608912	0.01194068
GATS1s	-0.62911365	0.39578399	0.01198345
vsurf_CW6	-0.62796234	0.39433671	0.01218818
SMR_VSA1	-0.62662334	0.3926568	0.0124297
AATSC0se	-0.62564902	0.39143669	0.01260775
vsurf_IW4	-0.62425279	0.38969155	0.01286635
ETA_dEpsilo	-0.62128127	0.38599042	0.01343041
ATSC0se	-0.61729156	0.38104887	0.01421765
vsurf_ID6	-0.61542145	0.37874356	0.01459871
DPSA5	-0.6145084	0.37762058	0.0147876
GATS4c	0.61417038	0.37720526	0.01485801
AATS1pe	-0.61379031	0.37673855	0.01493748
ATSC0pe	-0.61363801	0.37655161	0.01496942
ATSC1c	0.6135284	0.3764171	0.01499244
vsurf_HL2	-0.60837424	0.37011922	0.01610606
GATS6are	0.60807504	0.36975525	0.01617261
AATSC1c	0.60662344	0.367992	0.01649849
GATS6se	0.60550791	0.36663982	0.01675234
EState_VSA8	0.60496148	0.36597839	0.01687778
MINssCH2	0.603711	0.36446697	0.01716756
PEOE_VSA-6	-0.60192894	0.36231845	0.01758712

	r_pearson	r2	p_value
PEOE_VSA_F	-0.60192894	0.36231844	0.01758712
AATSC7i	0.60155067	0.3618632	0.01767718
AATS0se	-0.60030223	0.36036277	0.01797694
Q_VSA_PPOS	-0.59806477	0.35768147	0.01852392
AATSC0s	-0.59737757	0.35685996	0.01869445
ATSC1s	0.59676898	0.35613322	0.01884648
MATS7i	0.5949919	0.35401537	0.01929583
Mse	-0.59409478	0.35294861	0.01952577
MATS2se	0.59349774	0.35223957	0.01967995
vsa_pol	-0.59239338	0.35092992	0.01996761
AATSC1s	0.59128242	0.3496149	0.02026022
MATS8p	0.59097859	0.3492557	0.02034081
AATSC8p	0.59088547	0.34914564	0.02036556
Q_VSA_FPNE	-0.58954866	0.34756762	0.02072341
RNCS	-0.58836052	0.3461681	0.02104548
PEOE_VSA_F	-0.58737815	0.34501309	0.02131464
ATSC7i	0.58732586	0.34495167	0.02132904
PEOE_VSA_F	-0.58634934	0.34380555	0.02159934
PEOE_VSA_F	0.58634913	0.3438053	0.0215994
ATSC0s	-0.58547514	0.34278114	0.02184353
GCUT_SLOG	0.58398856	0.34104264	0.0222636
MATS8are	-0.58379599	0.34081776	0.02231846
vsurf_W6	-0.58313033	0.34004098	0.0225089
MATS2are	0.58311628	0.3400246	0.02251293
ATSC8p	0.58297663	0.33986176	0.02255305
GATS6pe	0.58134333	0.33796007	0.02302627
vsurf_HB1	-0.57808402	0.33418114	0.02399318
PEOE_VSA_F	-0.57582497	0.33157439	0.02468126
ZMIC1	0.57431272	0.32983511	0.02515017
vsurf_D7	0.57416598	0.32966657	0.02519603
GATS6s	0.57398556	0.32945942	0.0252525
vsurf_Wp4	-0.572074	0.32726866	0.02585672
vsurf_D8	0.57066625	0.32565996	0.02630867
AATS0are	-0.57049223	0.32546138	0.02636495
chi0_C	0.56919809	0.32398647	0.02678635
IC1	-0.56832963	0.32299857	0.02707199
SsOH	-0.5681269	0.32276818	0.027139
PEOE_VSA+	-0.56785491	0.3224592	0.0272291
PEOE_VSA_F	-0.56785491	0.3224592	0.0272291
nHBDon	-0.56785491	0.3224592	0.0272291
MATS8pe	-0.56494367	0.31916135	0.02820771
VSA_EState3	-0.5641704	0.31828824	0.02847206
MATS2pe	0.56352279	0.31755793	0.02869489
chi1v_C	0.5632337	0.31723221	0.02879478

	r_pearson	r2	p_value
GATS1dv	-0.561438	0.31521263	0.02942116
MATS1s	0.56014385	0.31376113	0.02987893
nC	0.55964921	0.31320723	0.0300553
GATS7i	-0.55865593	0.31209645	0.03041185
EState_VSA2	-0.5565865	0.30978853	0.03116491
MAXaaCH	0.55494481	0.30796374	0.03177224
density	-0.5547097	0.30770286	0.03185993
MID_C	0.55351938	0.30638371	0.03230674
MATS8se	-0.55332041	0.30616348	0.03238188
GATS8p	-0.55250144	0.30525784	0.03269256
AATS0s	-0.55127157	0.30390034	0.03316331
TASA	0.54946096	0.30190734	0.03386559
Mare	-0.54898016	0.30137921	0.03405394
BIC1	-0.54699463	0.29920312	0.03484007
EState_VSA9	-0.54311952	0.29497881	0.03641341
PNSA5	0.53933043	0.29087732	0.0380026
MINaaCH	0.53928134	0.29082437	0.03802352
MATS2s	0.53881488	0.29032148	0.03822276
AATS5se	-0.5387478	0.2902492	0.03825147
vsurf_ID8	-0.53831641	0.28978456	0.03843653
AMID_O	-0.53704929	0.28842194	0.03898396
vsurf_HB6	-0.53697036	0.28833717	0.03901825
AATSC7p	0.53560953	0.28687757	0.03961299
vsurf_R	0.53486134	0.28607665	0.03994285
h_emd	-0.53434526	0.28552485	0.04017156
AATS0pe	-0.53389923	0.28504839	0.04037002
ATSC7p	0.53356993	0.28469687	0.040517
MINaasC	0.53294726	0.28403279	0.04079602
AATS7p	0.53253159	0.28358989	0.04098307
MATS7p	0.53242441	0.28347575	0.04103141
vsurf_W2	-0.529017	0.27985899	0.04259025
nO	-0.52852751	0.27934133	0.04281774
MATS7v	0.52580347	0.27646928	0.04410025
vsurf_Wp5	-0.52337631	0.27392276	0.04526676
MINdssC	0.52106428	0.27150799	0.04639901
GATS2s	-0.52065734	0.27108406	0.04660044
GATS7v	-0.51948808	0.26986786	0.04718281
DCASA	-0.51931874	0.26969196	0.0472676
vsurf_CW2	-0.51911546	0.26948086	0.04736953
PM3_HF	0.51860268	0.26894874	0.04762736
AATSC7v	0.51841163	0.26875062	0.04772369
ATSC7v	0.51743375	0.26773769	0.04821899
vsurf_Wp3	-0.51706302	0.26735416	0.04840776
GATS1pe	-0.51548378	0.26572352	0.04921796

Table S2 220 chemical descriptors that significantly ($p<0.05$) correlate to selective toxicity against OSCC cells
